# A physics-informed neural network to model COVID-19 infection and hospitalization scenarios

**DOI:** 10.1186/s13662-022-03733-5

**Published:** 2022-10-27

**Authors:** Sarah Berkhahn, Matthias Ehrhardt

**Affiliations:** Applied and Computational Mathematics, Bergische Universität Wuppertal, Wuppertal, Germany

**Keywords:** Physics-informed neural networks, SIR, Compartment models, COVID-19, SARS-CoV-2, Epidemiology

## Abstract

In this paper, we replace the standard numerical approach of estimating parameters in a mathematical model using numerical solvers for differential equations with a physics-informed neural network (PINN). This neural network requires a sequence of time instances as direct input of the network and the numbers of susceptibles, vaccinated, infected, hospitalized, and recovered individuals per time instance to learn certain parameters of the underlying model, which are used for the loss calculations.

The established model is an extended susceptible-infected-recovered (SIR) model in which the transitions between disease-related population groups, called compartments, and the physical laws of epidemic transmission dynamics are expressed by a system of ordinary differential equations (ODEs). The system of ODEs and its time derivative are included in the residual loss function of the PINN in addition to the data error between the current network output and the time series data of the compartment sizes. Further, we illustrate how this PINN approach can also be used for differential equation-based models such as the proposed extended SIR model, called SVIHR model.

In a validation process, we compare the performance of the PINN with results obtained with the numerical technique of non-standard finite differences (NSFD) in generating future COVID-19 scenarios based on the parameters identified by the PINN. The used training data set covers the time between the outbreak of the pandemic in Germany and the last week of the year 2021.

We obtain a two-step or hybrid approach, as the PINN is then used to generate a future COVID-19 outbreak scenario describing a possibly next pandemic wave. The week at which the prediction starts is chosen in mid-April 2022.

## Introduction

To pursue the goal of developing a method for predicting future epidemiological trends, not only a numerical and a data-driven but also a mathematical approach are established to generate COVID-19 scenarios in this paper. All methods applied in this work to data on COVID-19-related population group sizes in Germany are also applicable to other countries for which data that can be transformed into sizes of model compartments is available.

The COVID-19 pandemic is currently one of the most discussed topics worldwide. The first cases of severe acute respiratory syndrome coronavirus type 2 (SARS-CoV-2) occurred in Asia in December 2019, but they were not reliably identified then. The People’s Republic of China experienced a peak of about 4600 cases per day in mid-February 2020, but by March 2020, the epidemic was largely contained in China and other Asian parts of the world. Europe experienced the first wave of the pandemic in March and April 2020, with, for example, about 5840 new daily infections in Germany in late March and 13,260 new daily infections in France in mid-April 2020.

While infection numbers in Europe were generally low in the summer of 2020, peaks were observed in the United States (∼67,000 infections/day), Brazil (∼46,000 infections/day), and India (∼93,000 infections/day) at certain times between July and September 2020. The third wave was characterized by approximately 35,000 new daily infections in Italy in mid-November 2020, 25,000 new daily infections in Germany around Christmas, 60,000 new daily infections in the United Kingdom in early January 2021, and severe lockdowns within Europe in fall 2020 and winter 2020/2021. The summer of 2021 was characterized by a relaxation of intervention measures in Europe. However, some countries experienced catastrophic COVID-19 events, such as India, with approximately 390,000 new daily infections in early May 2021 [16].

In November 2021, the fourth wave of the pandemic spread throughout Europe despite a fully vaccinated proportion of 67.6% in Germany, 68.9% in the United Kingdom, 74.8% in Italy, 77.7% in France, and 80.6% in Portugal [23]. Nearly 40,000 new daily infections were observed in Germany on November 14th 2021, as well as in the United Kingdom. Achieving even higher vaccination rates and providing booster vaccinations for all to maintain a high level of infection protection are policy issues of concern to all countries. The dangers posed by mutant virus variants such as the delta variant (B.1.617.2), which was first discovered in India in October 2020 and is now the dominant variant infecting people in several countries such as Germany, or the omicron variant (B.1.1.529) discovered in the autumn of 2021, are also being discussed in medicine and the literature. According to the Robert Koch-Institute (RKI), the mRNA vaccines from BioNTech/Pfizer, Moderna, and AstraZeneca are expected to have a protective effect of approximately 90% against severe infection with the alpha (B.1.1.7) variant and 75% against symptomatic infection with the delta (B.1.617.2) variant [20].

The mathematical model used in this work to describe the population dynamics of COVID-19 is an SVIHR model. It is based on a system of ordinary differential equations (ODEs). Most mathematical models describing the spread of the disease employ classical compartments in which the Susceptible-Infected-Recovered (SIR) structure is the most basic form of [2]. Mathematical modeling helps forecast the dynamics of infectious diseases. Over the past nearly two years, a variety of compartmental models have been introduced as enhanced SIR models to study various aspects of the COVID-19 pandemic.

It is clear that SIR models make simplifying assumptions about the population and disease process, which may be a reason to critically question and debate them. A study by Kharazmi et al. showed that a general disadvantage of COVID-19 models was the treatment of key parameter values as being fixed over time [10]. There are alternative methods to predict the course of the COVID-19 pandemic, including fitting curves to empirically observed data and solely data-driven non-numerical approaches. We always must keep in mind that the novelty of SARS-CoV-2 leads to many uncertainties in all modeling attempts due to biological features of transmission, viral mutations, pathogen behavior, and, of course, the unknown exact number of infections [10]. Moreover, no method can optimally predict the future, but a good model provides an approximation that is accurate enough to be useful for informing public policy [25]. The advantage of PINNs, which use physical laws governing the system in the form of equations, is the ability to be retrained as new data is collected and update the models over time with inferred parameters [10]. SIR-type models add a mathematical indication to a neural network, such that the exclusive data-drivenness is sustained with systematic knowledge of the disease transmission and behavior of the population. Depending on the number and kinds of used compartments and model parameters and their definition, an SIR-type model can be adapted to specific targets one has with respect to implementation. This can, for instance, be the estimation of future hospital occupancy, the influence of specific intervention measures, and isolation rates or the rate of asymptomatic infections. Respective transition rates can be fit into the model. The targets of the implementations and suitability of a certain model depend on the region or country used for data assimilation because of different conditions and disease spreads in distinct regions. In our approach, the country Germany is used for data collection and as the basis of model establishment, and our focus lies on infection and hospitalization numbers.

In this paper, we do not establish the simplest version of the compartment model. Instead, we develop some kind of extended model, which complements the basic SIR model by a vaccinated and a hospitalized class. The hospitalized compartment is added due to the high significance of hospitalization number predictions for hospital capacity planning and the assessment of the number of severely diseased individuals during pandemic times. We do not include an exposed compartment, which is a possible first enhancement to the SIR model and incorporates infected people who are not (yet) infectious, that is, those with pre-symptomatic and potentially asymptomatic individuals.

In our model, pre-symptomatic individuals are condensed with symptomatic people in the infected compartment, so that we have a single infected compartment of people not hospitalized. Since determining the proportion of asymptomatic individuals in the total infected population is not our goal at this point, we do not include a class of infected individuals who are asymptomatic, but assume at least very mild symptoms in infected individuals. The degree of infectivity of infected individuals can be controlled by adjusting the transmission rate in the model.

Our model includes a vaccination rate and the proportion of the population vaccinated each week. Therefore, the model is adaptable to different vaccination scenarios. In addition, the general transmissibilities of SARS-CoV-2 and its variants, which are constantly changing, lead to altered protective effects of available vaccines. The established model includes a transmission rate explained in Sect. 2.

The main method used in this work, called *Physics-Informed Neural Network* (PINN) and explained in Sect. 3.1, is used to estimate the transmission rate based on the data available in Germany. The PINN itself combines a data-driven method (here based on compartment size data, e.g., number of infections) with the developed ODE system so that it incorporates physical laws. In other words, this approach trades off between the data-based and physical loss functions in the training process. This reduces the space of feasible solutions to those that satisfy a ‘physical law’ to some degree, i.e., an SVIHR compartmental model in this case. The ODE system corresponding to the model serves as an additional constraint in the training phase encoded by an appropriate additional residual loss term. More specifically, the PINN loss function consists of the two weighted terms *data loss* and *residual loss*. The data loss is calculated as the difference between the current network output in terms of infection or hospitalization numbers and the reported 2019 coronavirus pandemic (COVID-19) data covering weeks between March 2020 and December 2021. The residual loss is based on a mathematical model with a system of ordinary differential equations that describes the main population dynamics observed during the COVID-19 pandemic. A PINN approach for the simple SIRD model was proposed by Malinzi et al. [11], and a PINN approach for an SIR-based vaccination model was described by Torku et al. [26]. In contrast, Zeroual et al. [28] compared different pure deep learning models for forecasting COVID-19 cases and found the Variational AutoEncoder (VAE) algorithm to be superior.

Raissi et al. [17] explain that PINNs are neural networks that embed physics as a regularization term in the loss function. They say that given a sufficient number of data points and an expressive neural network architecture, they can achieve good approximation accuracy if the given differential equation is well-posed and has a unique solution. PINNs can also be viewed as a *surrogate model* for solving differential equations by incorporating additional data or as a data-driven correction (or even discovery) of the underlying physical system. One motivation for this hybrid approach can be seen in the observed non-compliance of some individuals with social distancing (or physical distancing) and hygiene rules. This behavior is difficult to formulate in ODEs, but it is included in the neural network training data.

Olumoyin et al. [15] use the term *Epidemiology-Informed Neural Network (EINN)*, which describes a type of feedforward neural network that incorporates epidemiological dynamics such as lockdown into its loss function. Their EINN learns solutions for the so-called asymptomatic SIR model, i.e., the proportion of asymptomatic infected individuals to the total number of infected individuals.

Shaier et al. [22] use the term *Disease-Informed Neural Networks (DINN)* to refer to a type of PINN-based neural network that can be applied to increasingly complex systems of differential equations describing various known infectious diseases. The DINN formulation learns both the representation of the underlying system as a neural network and also performs a calibration (called ‘Inverse Parameter Estimation’) for the assumed ODE system model. In this way, it can be used to predict the infection rates, etc.

As our PINN operates based on transmission and transition dynamics in a population affected by COVID-19, estimates transmission rate parameters, and incorporates a transmission rate that can incorporate remedial measures, such as quarantine and contact restrictions, our PINN can be described as a special type of EINN designed to predict COVID-19 incidence. Because our PINN uses a system of differential equations to learn the parameters that generate it, this PINN can also be considered a DINN. Our approach of using PINN-identified parameters of an ODE system to predict infection and hospitalization rates by using the PINN itself in a slightly modified form and a numerical method of NSFD is innovative. The uniqueness of our approach lies in the fact that we use our PINN for parameter identification and give it a second input of initial compartment size data to generate accurate future compartment size scenarios, and then apply a purely numerical method so that we can compare the predictions of the data- and ODE-based PINN with their predictions.

The data used consist of infection and hospitalization rates as well as vaccination, death, and cure rates for Germany obtained from the RKI [18, 19]. The PINN also works based on the established dynamic ODE system, which forms the core of the model and is developed in Sect. 2. The transmission rate is one of the most important parameters affecting the occurrence of infections and thus the established ODE system. Therefore, changes in transmissibility due to mutations or altered susceptibility of the underlying population are part of the model-based predictions.

The exact procedures used in this work are described in Sect. 3.2 and Sect. 3. In this work, the estimation of certain model-specific parameters is performed by the PINN mentioned above. A model-specific non-standard finite difference scheme (NSFD) is a numerical integration method for comparison and validation.

The data on which our implementations are based covers the calendar weeks 10 in 2020 through 14 in 2022, obtained from the inquiries of the German Robert-Koch Institute (RKI). Different weights for the loss terms are used as examples in the prediction section to analyze the impact of weight modification.

## Model structure

In this work, an SVIHR compartmental model was developed based on the basic SIR model introduced by Kermack and McKendrick in 1927. The SIR model consists of three compartments of susceptible (*S*), infected (*I*), and recovered (*R*) individuals. Susceptible individuals have not yet become infected but may become ill. Infected individuals have already become infected. In the basic SIR model, they can also infect susceptible persons. Therefore, they are assumed to be infectious and may or may not have symptoms. Recovered individuals have overcome the disease and are no longer ill.

### The SIR model in epidemiology

The basic SIR model assumes that no births or deaths enter the system, that the population is closed so that no one enters or leaves a compartment from the outside, and that recovered individuals are completely immune so that they can never be reinfected. The total size of the population at a time *t* is denoted by $N(t)$. The satisfaction of the equation

$$ N(t) = S(t)+I(t)+R(t) \quad \text{with } N\colon [0,T]\to \mathbb{N}, $$

means that the number of individuals in the system is the sum of the compartment sizes at each time point considered $t\in [0,T]$. The system must have initial conditions $S(0)$, $I(0)$, $R(0)$ to be well-defined [12, p. 11]. The population size $N(t)$ is constant if the derivative of $N(t)$ is zero. If there is no natural death rate and no recruitment or birth rate in the system, or if the natural death rate and recruitment rate equilibrate, this constancy is given. The individuals in the system are infected, i.e., they migrate from compartment *S* to *I* at a rate $\theta (t)$, which is defined as

1 $$ \theta (t) := \beta \gamma (t) (1-q ) I(t) , $$

where *β* is the transmission risk, and $\gamma (t)$ is a time-dependent contact rate. The parameter *q* symbolizes the degree of strength of intervention, quarantine, and isolation measures implemented. For example, when more infectious individuals are isolated, fewer further infections occur. The rate

2 $$ \Theta (t) := \theta (t) \frac{S(t)}{N(t)} $$

is called a standard incidence rate, and $\theta (t)$ is the force of infection and is called the transmission rate.

### The SVIHR model

The basic model is extended in this work to include a vaccinated compartment *V* and a hospitalized compartment *H*. Infected individuals remain infected for $T_{I}$ days until they recover, when a proportion *ξ* of all transiting individuals are hospitalized. Thus, the rate $\omega _{1}$ at which persons per unit time (week) pass from compartment *I* to *R* is given by

3 $$ \omega _{1} = \frac{1-\xi}{T_{I}}, $$

and the rate *η* at which individuals are reach the compartment *H* per unit of time is defined as

4 $$ \eta = \frac{\xi}{T_{I}}. $$

Hospitalized individuals are assumed to infect susceptible individuals only to a neglectable degree due to their isolated state and good hygienic precautions. They remain infected for $T_{H}$ days from the time of their hospitalization. A proportion $\mathcal{M}$ of the individuals transiting from the compartment *I* to *H* is assumed to die from disease-related causes. The rate $\omega _{2}$ at which persons per unit time pass from compartment *H* to *R* is given by

5 $$ \omega _{2} = \frac{1-\mathcal{M}}{T_{H}}. $$

Susceptibles reach the compartment *V* at a rate $\mathcal{V}$. The condition for the transition from the compartment *S* to *V* is receiving a second vaccination against SARS-CoV-2. Since vaccination does not guarantee complete immunity to infection, i.e., we speak of a *leaky vaccination*, it is assumed that vaccinated individuals in the system may contract the infection with a small probability. The respective rate at which vaccinated individuals pass into the infected compartment *I* is $\kappa \theta (t)$, where *κ* denotes the residual probability of infection after vaccination.

BioNTech/Pfizer’s Comirnaty and Moderna’s Spikevax vaccines are about 95% effective, AstraZeneca’s Vaxzevria vaccine is about 80% effective, and Johnson & Johnson’s Janssen vaccine is about 65% effective. Thus, a leaky-vaccinated compartment is assumed rather than an all-or-nothing vaccinated compartment. Because leakiness was assumed, all vaccinated individuals have a lower probability of contracting the infection than susceptible individuals in compartment *S*. When an all-or-nothing vaccine was assumed, vaccination provided complete protection from infection to a portion $\mathcal{V}$ of the susceptible class per unit time *t*, whereas the $1-\mathcal{V}$ portion received no protection.

Finally, we include a constant system inflow, the birth rate Λ (e.g., birth of new individuals that can get infected, and the natural mortality rate *μ*). A constant rate $\theta =\beta \gamma (1-q) $ is used in the implementations of the PINN. The corresponding system of ordinary differential equations (ODEs) has the following form in which we set $\gamma =1$ and $q=0$:

6 $$ \begin{gathered} \frac{\mathrm{d}S(t)}{\mathrm{d}t} = \Lambda - \beta \frac{S(t)}{N(t)} - (\mathcal{V}+\mu ) S(t), \\ \frac{\mathrm{d}V(t)}{\mathrm{d}t} = \mathcal{V} S(t) - \beta \kappa \frac{S(t)}{N(t)}-\mu V(t), \\ \frac{\mathrm{d}I(t)}{\mathrm{d}t} = \beta (1+\kappa ) \frac{S(t)}{N(t)} - (\eta + \omega _{1} +\mu ) I(t), \\ \frac{\mathrm{d}H(t)}{\mathrm{d}t} = \eta I(t) - (\omega _{2}+\mu ) H(t), \\ \frac{\mathrm{d}R(t)}{\mathrm{d}t} = \omega _{1} I(t) + \omega _{2} H(t)-\mu R(t). \end{gathered} $$

The total population is now defined as

$$ N(t)=S(t)+V(t)+I(t)+H(t)+R(t). $$

Since the pandemic has a faster dynamic than the birthrate and the natural mortality, $N(t)$ can be regarded as a conserved quantity of the above ODE system if we set Λ and *μ* to zero.

**Figure 1:**
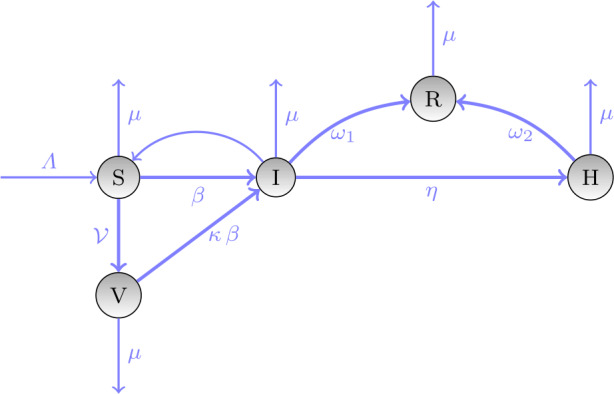
Compartment model for the SVIHR model

The dynamical system described by equation (6) is depicted in Fig. 1. Blue arrows from one compartment to another indicate a transition, where the compartment from which a red dashed arrow emanates can infect susceptibles.

## Methods

Data were obtained from the Robert Koch-Institute (RKI) [18, 19] and the German COVID-19 vaccination dashboard [4]. They refer to the calendar week 10 in 2020 through 14 in 2022. Our validation was based on the calendar weeks 10 in 2020 through 52 in 2021. The first omicron wave initially emerged in October 2021 and lasted until the end of December 2021 [16]. Since the omicron variant was responsible for high infection, reaching a peak of almost 3000 daily infections per 1 million people on March 31st 2022 [16] and our PINN should be adapted to this variant, the first omicron wave was included in the data of our validation runs. A local maximal incidence of 691 daily infections per 1 million inhabitants was attained on December 1st 2021 [16]. Weekly case-hospitalization, case-fatality, and vaccination rates were computed on the basis of the given data sets. The RKI registers deceased individuals who have been identified as having the SARS-CoV-2 pathogen as those who died from COVID-19. In Sect. 3.1, the approach of PINN is explained. Section 3.2 explains the technical procedure of building the PINN. In Sect. 3.3, the technique of Nonstandard Finite Difference Schemes (NSFD) is explained.

### Physics-informed neural networks for compartment models

The basic concept of *physics-informed neural networks* (PINN) is to incorporate the laws of dynamical systems modeled by ordinary or partial differential equations into a deep learning framework. The loss function of the corresponding neural network includes not only solely the so-called *loss error* related to the difference between the output of the network and the reported data used but also the so-called *residual error* related to the ODEs or PDEs. The sum of these two errors is then minimized in the least squares sense.

The weighted loss function consists of the data loss and the residual loss term. As pointed out in [9], the training using the data loss (i.e., measurements, physics-uninformed) is regarded as supervised learning while the training w.r.t. the residual loss using the governing differential equation (physics-informed) is regarded unsupervised learning.

Let the vector *ϑ* of all parameters included in (6) be given by

7 $$ \vartheta =[\beta , \gamma , q, \mathcal{V}, \kappa , \xi , T_{I}, T_{H}, \mathcal{M}, \Lambda , \mu ]^{\top} . $$

The parameters in *ϑ* can be partitioned into fixed parameters $p_{f}$ and trainable parameters *p*, which we select as follows:

$$ \begin{gathered} p_{f} := [\gamma , q, \mathcal{V}, \xi , T_{I}, T_{H}, \mathcal{M}, \Lambda , \mu ]^{\top }, \\ p := [\beta , \kappa ]^{\top} . \end{gathered} $$

We selected *β* and *κ* as the trainable parameters in the network because no reliable or clear values for them could be found in studies.

Our PINN

$$ \mathcal{PINN}^{W}_{p}\colon \mathbb{R}^{+} \to \mathbb{R}^{n} $$

is used to approximate the solution

8 $$ \mathcal{K}_{p}=[S,V,I,H,D,R]^{\top}\colon \mathbb{R}^{+}\to \mathbb{R}^{n} , $$

of the system of ODEs (6) by performing error minimization during training [7]. The superscript *W* symbolizes the weights used during the forward and backward propagation in the neural network. The subscript *p* represents the model parameters. At time instance *t* the solution is expressed as

$$ \mathcal{K}_{p}(t)= \bigl[\mathcal{K}_{p}^{1}(t), \dots ,\mathcal{K}_{p}^{n}(t) \bigr]^{\top } , $$

where $\mathcal{K}_{p}^{d}(t) \in \mathcal{C}^{1}(\mathbb{R})$ is the output of the PINN for the *d*th compartment at time *t*. The parameters *W* and *p* are optimized during the backpropagation process of the neural network such that $\mathcal{PINN}_{p}^{W}$ fits the reported data $\hat{\mathcal{K}}$ in a least-squares sense [7].

Scalar time instances $t_{j}$ are fit as input to our network. Let

$$ \hat{\mathcal{K}}^{j}=\bigl[\hat{\mathcal{K}}^{1,j},\dots , \hat{\mathcal{K}}^{n,j}\bigr]^{\top}, \quad j \in \{1,\ldots ,l\} $$

be the vector of the reported sizes of those *n* compartments at time instance $t_{j}$. If $\mathcal{K}$ denotes the *n* compartments, we express the terms on the right-hand side of (6) by

$$ F_{p}(\mathcal{K})= \bigl[F_{p}^{1}( \mathcal{K}),\dots ,F_{p}^{n}( \mathcal{K}) \bigr]^{\top } , $$

where *n* is the number of compartments, i.e. $n=5$ in the case of the SVIHR model. It holds that $F_{p}^{d} = F_{p}^{d}(\mathcal{K}(t)) \in \mathcal{C}(\mathbb{R}^{5})$, for all $d\in \{ 1,\dots ,n \}$. The system of ODEs in (6) have the following form

9 $$ \frac{d\mathcal{K}(t)}{dt}-F_{p}(\mathcal{K})=0, \quad j \in \{1, \dots ,l\} . $$

We define $T:=[t_{1},\ldots,t_{l}]$ as the vector of input time instances. We obtain one output of the PINN per training iteration. Per *k*th training iteration $Z_{k}$, $k \in \{1,\dots ,M\}$, we compute the usual *data error* defined as

10 $$ \mathrm{MSE}_{\mathcal{U}} =\mathrm{MSE}_{\mathcal{U}}(W,p,Z_{k}) := \frac{1}{l}\sum_{j=1}^{l} \bigl\Vert \mathcal{PINN}_{p}^{W,Z_{k}}(t_{j})-\hat{ \mathcal{K}^{j}} \bigr\Vert ^{2} . $$

Next, let us extend the loss function of the PINN by the additional term

11 $$ \mathcal{F}_{p}\bigl(\mathcal{PINN}_{p}^{W},t_{j},Z_{k} \bigr) := \frac{d\mathcal{PINN}_{p}^{W,Z_{k}}(t)}{dt}\bigg|_{t=t_{j}} - F_{p} \bigl( \mathcal{PINN}_{p}^{W,Z_{k}}(t_{j}) \bigr) , $$

where

12 $$ \mathcal{F}_{p}\bigl(\mathcal{PINN}_{p}^{W},t_{j},Z_{k} \bigr) = 0 \quad \text{for all } j \in \{1,\dots ,l\} $$

means that the PINN satisfies the given system of ODEs in a certain discrete time grid (similar to the collocation method).

The time derivative of the neural network output $\frac{d\mathcal{PINN}_{p}^{W,Z_{k}}(t)}{dt} |_{t=t_{j}}$ can be computed using automatic differentiation [1]. We obtain the physics-informed part of the loss function, the *residual error*, per training iteration as

13 $$ \mathrm{MSE}_{\mathcal{F}} = \mathrm{MSE}_{\mathcal{F}}(W,p,Z_{k}) := \frac{1}{l}\sum_{j=1}^{l} \bigl\Vert \mathcal{F}_{p}\bigl(\mathcal{PINN}_{p}^{W,Z_{k}},t_{j} \bigr) \bigr\Vert ^{2} . $$

We introduce a hyperparameter and weighting factor $\alpha \in [0,1]$. They weight the data loss and residual loss in the loss function. Per training iteration, a loss is computed. The loss function of the PINN is defined as

14 $$ \mathcal{L}_{\alpha }=\mathcal{L}_{\alpha}(W,p,Z_{k}) :=\alpha \mathrm{MSE}_{ \mathcal{U}} + (1-\alpha ) \mathrm{MSE}_{\mathcal{F}} . $$

The minimization problem of the neural network is then given by

15 $$ \min_{W,p} (\mathcal{L}_{\alpha } ) . $$

### Procedures of building the PINNs

A single feed-forward PINN was used for each infected compartments *I* and *H*. This was done so that separate parameter vectors $p:=[\beta ,\kappa ]^{\top}$ were estimated per run of the neural network for *I* and *H*. Among all model parameters, it is the most difficult to assign realistic values to *β* and *κ* from raw data. The recruitment and natural death rate are set to zero as they are regarded as equal here, but are still included in the system of ODEs to be able to derive the denominator function in the NSFD scheme, see Sect. 3.3. Values for the case-fatality, the case-hospitalization, and the vaccination rate were computed from the available RKI data [18, 19]. The fixed model parameters were computed as $\mathcal{V}=0.013517486$, $\xi =0.079718848$ and $\mathcal{M}=0.026720524$ from the given data sets [18, 19]. According to the RKI, contagiosity strongly recedes after a mean of 10 days of infectedness [21]. In an article on hospitalization of COVID-19 cases compared to flu epidemics, the mean duration of COVID-19-induced hospitalization in Germany was 10 days, while the length of stay in hospital for people transferred to the intensive care unit was 16 days, and the hospital sojourn time for persons on mechanical ventilation, on average, was 18 days [24]. We set the parameter concerning the length of stay in the infected state to $T_{I}=1.42$ weeks and used a slightly higher value of $T_{H}=1.5$ weeks for the hospital sojourn time in our implementations. We selected the transmission rate *β* and the transmission variation coefficient for the vaccinated *κ* as trainable parameters. In further implementations or using a different model, other or more model parameters could be selected as trainable.

We used three hidden linear layers with 5 neurons each for the parameter identification part and 6 hidden layers with 350 neurons each for the compartment size prediction part of the PINN. A linear output layer was applied to obtain a compartment size output, and a ReLU output layer was used for the trained parameter vector. ReLu or tanh functions were used as activation functions per layer. The Adam algorithm was selected as the optimizer. The used learning rate was $\mathit{lr}=0.003$. Different layers and activation functions were tested and compared with respect to output compartment size curves. The selected ones yielded the most reasonable size ranges. The ReLU output layer was used for the parameter vector prediction, particularly because non-negative values were wanted. To compute the derivative $\mathrm{d}{\mathcal{PINN}_{p}^{W}(t)}/\mathrm{d}t$ the PyTorch automatic differentiation package torch.autograd.grad was used. It computes and returns the sum of gradients of the respective compartment size tensor $\mathcal{PINN}_{p}^{W}(t)$ with respect to the input time tensor *t*. Results of the compartment size and trainable parameter vector predictions of the PINN are described in Sect. 4.

Subsequently, the prognosticated parameters *β* and *κ* were used as the inputs to a nonstandard finite difference (NSFD) scheme in a validation process. NSFD schemes are explained in Sect. 3.3. They preserve certain properties like the positivity or the asymptotic behavior of the analytic solution of differential equations on the discrete level. Their most important characteristic is, in many cases, the complete absence of the elementary numerical instabilities which plague common finite difference schemes [13].

In the whole validation procedure, the errors between the predictions obtained through these two methods and the actual available data were analyzed and compared. The corresponding results can be found in Sect. 4.1. For the PINN implementations the PyTorch Library was used.

### Nonstandard finite difference schemes

NSFD methods for the numerical integration of differential equations had their origin in a paper by Mickens published in 1989 [13]. In this section, an NSFD scheme is constructed to satisfy the essential positivity condition and the conservation law for $\Lambda =\mu =0$ which leads as a byproduct to the stability of the scheme. The interested reader may also check that the equilibrium points of the ODE model also appear in the proposed NSFD-scheme. We recall that schemes such as those based on Runge-Kutta methods [5] can produce ‘false’ or ‘spurious’ fixed-points, which are not fixed points of the original ODE system, cf. [14]. Finally, we will determine the so-called denominator function, such that we obtain the correct long-time behavior. We refer to [27], where we established an NSFD scheme for a similar compartment model as here. We implemented a simultaneous parameter estimation using a nonlinear least squares minimization of the error between time series compartment size data and the result of the NSFD-based integration of the respective system of ODEs. This does not equal the data or residual loss of our PINN approach used in this paper. With the optimized parameters and the NSFD scheme, we generated future COVID-19 scenarios. Here, we are now able to compare NSFD results to the results obtained using neural networks.

A numerical scheme for a system of first-order differential equations is called NSFD scheme if at least one of the following conditions [13] is satisfied:

- The orders of the discrete derivatives should be equal to the orders of the corresponding derivatives appearing in the differential equations.
- Discrete representations for derivatives must, in general, have nontrivial denominator functions. Here, the first-order derivatives in the system are approximated by the generalized forward difference method (forward Euler method) $\frac{\mathrm{d}u_{n}}{\mathrm{d}t} \approx \frac{u_{n+1}-u_{n}}{\phi (h)}$, where $u_{n}\approx u(t_{n})$ and $\phi \equiv \phi (h)>0$ is the so-called *denominator function* such that $\phi (h)=h+\mathcal{O}(h^{2})$, with *h* the step size. This function $\phi (h)$ is chosen so that the discrete solution has the same asymptotic behavior as the analytical solution.
- The nonlinear terms are approximated by non-local discrete representations, for instance, by a suitable function of several points of a mesh, like $u^{2}(t_{n}) \approx u_{n} u_{n+1}$ or $u^{3}(t_{n}) \approx u^{2}_{n} u_{n+1}$.
- Special conditions that hold for either the ODE and/or its solutions should also apply to the difference equation model and/or its solution, e.g., positivity of the solution, convexity of the solution (in finance), equilibrium points of the ODE system, including their local asymptotic stability properties.

In NSFD schemes, derivatives must be modeled by discrete analogues that take the form, cf. [13]

16 $$ \frac{\mathrm{d}u(t)}{\mathrm{d}t} \to \frac{u_{n+1}-\psi (h)u_{n}}{\phi (h)} , $$

where $t_{n}=n h$, $u_{n}$ is the approximation of $u(t_{n})$, and $\psi (h) = 1 + \mathcal{O}(h)$. The purpose of this more general time discretization (16) in NSFD schemes is to properly model the asymptotic long-time behavior of the solution.

Next, we propose the following NSFD discretization for solving the ODE system (6) 17 with a denominator function $\phi (h)$ to be determined later, given by (26). Let us briefly comment on the discretizations of the nonlinear (here: quadratic) terms. For example, in the first line (17), we have discretized the nonlinear contact term $\beta I(t)S(t)$ in (6) by $\beta I^{n} S^{n+1}$ rather than, say, $I^{n} S^{n}$ or $I^{n+1} S^{n+1}$. The rule is that exactly one factor of the variable appearing in the time derivative (here *S*) must be taken at the new time level $n+1$. This is needed to obtain a positivity preserving scheme, see (18). Not to destroy the explicit sequential evaluation, all other variables are taken from the previous time level, unless they are already known from a previous step, like $I^{n+1} S^{n+1}$ in the third line. If possible, discrete conservation properties (here: total population) must also be taken into account.

Observe that although the initial scheme (17) can be considered implicit, the variables at the ($n+1$)-th discrete-time level can be explicitly calculated in terms of the previously known variable values as given in the sequence of the equations above, i.e. we can rewrite it as an explicit form 18 The calculation must be done in exactly this order. All parameters appearing in these type of epidemic models are always non-negative. This is the convention used in fields related to the spread of diseases. From the explicit representation (17), it is easy to deduce that this scheme preserves the positivity, given some natural conditions on the parameters.

Finally, it only remains to correctly determine the denominator function $\phi (h)$. To do so, we reconsider the total population $N=S+V+I+H+R$ of the ODE system (6), now without neglecting Λ and *μ*. Adding the equations of (6), we easily obtain the following differential equation describing the dynamics of the total population *N*

19 $$ \frac{\mathrm{d}N(t)}{\mathrm{d}t} = \Lambda -\mu N(t) . $$

It is solved by

20 $$ N(t) = \frac{\Lambda}{\mu} + \biggl(N(0)-\frac{\Lambda}{\mu} \biggr) e^{- \mu t} = N(0) + \biggl(N(0)-\frac{\Lambda}{\mu} \biggr) \bigl(e^{-\mu t}-1\bigr), $$

with $N(0)=S(0)+V(0)+I(0)+H(0)+R(0)$. From (20), we immediately deduce that we have in the long term $\lim_{t\to \infty}N(t)=\Lambda /\mu $. Let us briefly note that this link between the transient dynamics and their ‘natural’ limiting systems can be used to reduce the dimension of this model, cf. [3]. Next, adding the equations in the discrete NSFD model (17) yields

21 $$ \frac{N^{n+1}-N^{n}}{\phi (h)} = \Lambda +\beta (1+\kappa ) \bigl(I^{n+1}-I^{n}\bigr)S^{n+1} - \mu N^{n+1}, $$

and for the purpose of studying the asymptotic behavior, we assume that *I* is stationary, $I^{n+1}=I^{n}$, such that the second term in (21) vanishes:

22 $$ \frac{N^{n+1}-N^{n}}{\phi (h)} = \Lambda - \mu N^{n+1}, $$

i.e.

23 $$ \begin{aligned} N^{n+1}&= \frac{N^{n} +\phi (h)\Lambda}{1+\phi (h) \mu} =N^{n}- \biggl(N^{n} -\frac{\Lambda}{\mu} \biggr) \frac{\phi (h) \mu}{1+\phi (h) \mu} \\ &=N^{n}+ \biggl(N^{n} -\frac{\Lambda}{\mu} \biggr) \biggl( \frac{1}{1+\phi (h) \mu}-1 \biggr). \end{aligned} $$

The denominator function can be derived by comparing Equation (22) with the discrete version of Equation (20), that is

24 $$ N^{n+1} = N^{n} + \biggl(N^{n}- \frac{\Lambda}{\mu} \biggr) \bigl(e^{-\mu h} - 1\bigr), \quad h=\Delta t, $$

such that the (positive) denominator function is defined by

25 $$ \frac{1}{1+\phi (h) \mu}=e^{-\mu h}, $$

i.e.

26 $$ \phi (h) = \frac{e^{\mu h}-1}{\mu} = \frac{1+\mu h + \frac{1}{2} \mu ^{2} h^{2} + \cdots - 1}{\mu} = h + \frac{\mu h^{2}}{2} + \cdots = h + \mathcal{O}\bigl(h^{2}\bigr). $$

Note that the conservation property requires all the denominator functions $\phi (h)$ for the compartments to be the same.

## Results

The results are separated into the validation of the PINN for infection and hospitalization number prediction, given in Sect. 4.1 and Sect. 4.2. A prediction of infection numbers of our PINN for different assignments of the parameter weighting the two loss terms is provided in Sect. 4.3.

The trained PINN identifies the parameters *β* and *κ*. The data set $\hat{\mathcal{K}}=[\hat{S},\hat{V},\hat{I},\hat{H},\hat{R}]^{\top}$ is used in the loss computation. The wanted vector of trainable parameters obtained from the trained neural network varied between different test runs. The size of the respective compartment *I* or *H*, the size of which is obtained as a second output of the PINN, partly depends on the architecture of the network, that was improved during the validation process. Values of the two trainable parameters obtained from PINN are later used as the inputs to further Python codes used for the formation of future SARS-CoV-2 scenarios. In the following, the term *training iteration* is used for one step with loss computation and parameter update, and a *training* refers to one training epoch containing multiple training iterations.

### Validation process: infection rates

At first, we investigated what values the PINN estimated for the two trainable parameters. The PINN identified $\kappa \in [0.0005, 0.0015]$ and $\beta \in [0.07 \cdot 10^{-8}, 0.52 \cdot 10^{-8}]$ within 200 trainings if only 5000 training iterations were used. If 20,000 training iterations were used, the PINN identified $\kappa \in [0.0009, 0.0011]$ and $\beta \in [0.19 \cdot 10^{-8}, 0.22 \cdot 10^{-8}]$ within another 200 trainings. The decrease in the identified interval shows a positive effect of the increase in the number of training iterations on the performance of the PINN in terms of parameter identification. As input to the NSFD scheme, we used $\kappa =0.001$ and attempted $\beta \in [0.18 \cdot 10^{-8}, 0.22 \cdot 10^{-8}]$ to investigate and compare resulting curves with the reported data.

**Figure 2:**
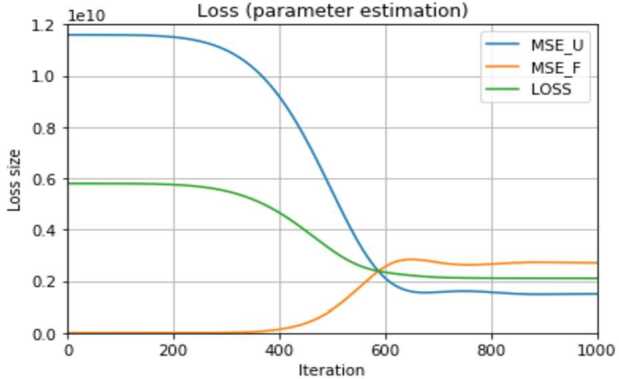
Trends of the errors $\mathrm{MSE}_{\mathcal{U}}$, $\mathrm{MSE}_{\mathcal{F}}$, and the loss $\mathcal{L}_{0.5}=\frac{1}{2}\mathrm{MSE}_{\mathcal{U}}+\frac{1}{2}\mathrm{MSE}_{ \mathcal{F}}$ in 1000 observed training iterations of the PINN for the estimation of *β* and *κ*

Figure 2 shows the loss obtained in a run of the PINN for the estimation of the trainable parameters.

It can be seen that the data loss $\mathrm{MSE}_{\mathcal{U}}$, computed as the mean squared error between reported and network-generated compartment size data using the Python function *MSELoss*, decreases by more than 90% between the first and the 640th training iteration. This means that the updated sizes of the compartment *I* approaches the reported infection numbers by weight updates of the neural network during training in these iterations. Then, it remains on a level of approximately $0.17\cdot 10^{9}$. The residual loss $\mathrm{MSE}_{\mathcal{F}}$ increases from 1.079 to a maximal value of $2.838 \cdot 10^{9}$ during the same number of iterations, then is very marginally reduced to around $2.710 \cdot 10^{9}$, and remains on this level. The training loss consequently decreases from $5.8 \cdot 10^{9}$ in the beginning to $2.2 \cdot 10^{9}$ in the 640th training pass, remaining on this level, and was thus reduced by 62%. The obtained sizes of the loss, $\mathrm{MSE}_{ \mathcal{U}}$ and $\mathrm{MSE}_{\mathcal{F}}$ over the course of several iterations of one training depend, among others, on the size of *α*, but a decreasing loss was observed in all performed trainings with different *α* and for both the prediction of infection and hospitalization numbers. In Fig. 2, the *x*-axis contains only 1000 iterations to put the focus on the initial behavior of the three functions, but we actually used 20,000 iterations in the following validation part.

Secondly, we conducted a validation by measuring how close the prediction of our PINN with different assignments of the weighting parameter *α* approximated the reported data. The target was to find out which weighting of the data and residual loss in the loss function results in the best approximation, as well as to generally evaluate the suitability of our PINN for predictions.

Our PINN uses labeled training data, i.e., compartment sizes $\hat{\mathcal{K}}$ assigned to specific weeks $t_{i}$, $i \in \{1,\dots ,l\}$, in every training iteration. As training data are not unlabeled here, we cannot describe our PINN approach as an unsupervised neural network. Nonetheless, our PINN is not a typical kind of supervised classification network but learns the course of an infectious disease in a data- and model-driven way. As a consequence, our validation does not consist of a sensitivity or specificity analysis, which is typically conducted for supervised neural networks. We focus on the investigation of the predicted trends with their local maxima and minima during the course of the pandemic from August 2021. We also compute errors to be able to more specifically compare the results obtained with different weighting parameter values.

The time period that predictions refer to, which is 27 or 35 calendar weeks here, always has to be considered. A predicted time period of half or two-thirds of a year is reasonable with respect to COVID-19 scenarios because it covers approximately one large wave of the pandemic, which may have several local peaks and usually has one global peak. So in the context of COVID-19, the term *long-term prediction* can be associated with the prediction of one following wave that is dominated by infections with a specific variant of the virus. A *short-term prediction* can be described as a forecast of e.g. the next local peak within a wave then. Another interpretation of a *short-term prediction* is a forecast based on certain most current data, which could be data referring to the last experienced wave or high peak during the pandemic. In our following validation, we do not restrict the training data to the most recent peak of the omicron wave but still incorporate all three experienced peaks during the omicron wave into our training data. A corresponding aim is that the PINN shall be able to foresee peaks in the height of the ones substantially caused by omicron infections and is simultaneously adapted to various virus variants.

It is also significant to consider the data set with which the training procedure works. Depending on different viral mutations and also state intervention or compliance of the population during the pandemic, the underlying data set varies, certain parameters in the system of ODEs must be adapted, and possible predictions change. For instance, the high infection rates during the B.1.1.529 (omicron) wave with 58,107 daily new infections on November 28th 2021 (local maximum), 192,396 daily new infections on February 10th 2022 and 251,509 daily new infections on March 31st 2022 (global maximum) cannot be foreseen if not enough weeks in which the omicron variant was dominant are included [16]. We incorporated data reaching until the end of the year 2021, which was after the first COVID-19 wave with a dominant omicron variant.

**Figure 3:**
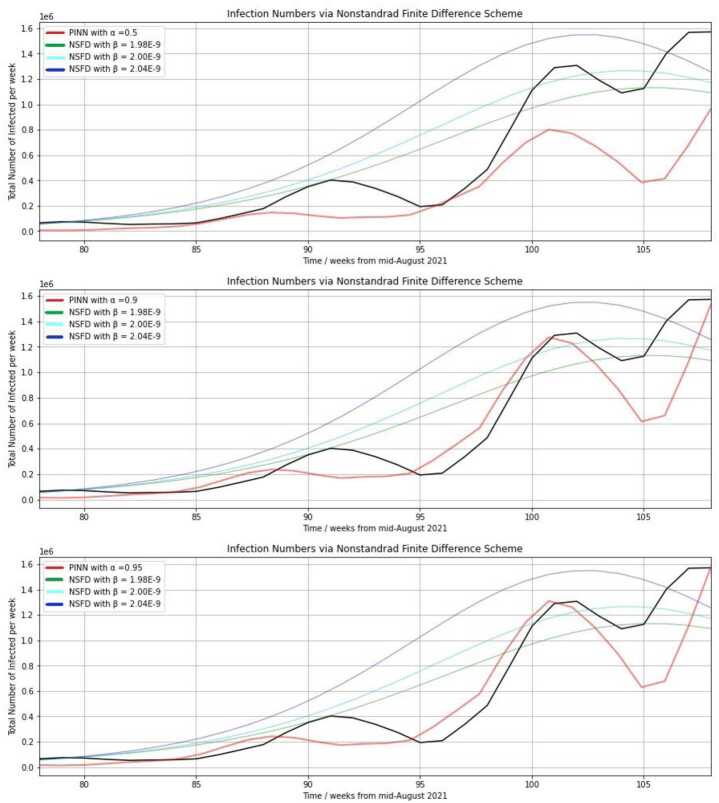
Infection numbers obtained from the reported data (black), the training of the PINN (red) or the NSFD scheme with distinct transmission risk parameter values (blue, cyan, green) from the 78th regarded week (34th calendar week in 2021) for three different assignments of the weighting parameter *α* ($\alpha = 0.5, 0.9, 0.95$). The used training data cover the calendar weeks 10 in 2020 through 52 in 2021 (1st through 96th regarded week)

Figure 3 shows the weekly infection rates published by the RKI and the respective prediction of the PINN for three different choices of the weighting parameter *α* in three different diagrams. In all three plots, three forecasts obtained from using the NSFD scheme based on three different choices of the transmission risk parameter *β* are included. The black curve is the same in the three diagrams below and reflects the reported data. The same NSFD predictions are shown in all four diagrams of Fig. 3, but a distinct PINN prediction is depicted in each diagram.

In Fig. 3, the points in time at which real local peaks (black curve) were attained are the 91st regarded week (calendar week 47, 2021) with 403,329 weekly new infections, the 102nd regarded week (calendar week 6, 2022) with 1,307,475 weekly new infections and the 108th regarded week (calendar week 12, 2022) with 1,571,595 weekly new infections. We note that the choice of the parameter *β* has a considerable effect on performance of the NSFD scheme. In the approximation of the NSFD scheme, a larger *β* leads to a higher and earlier reached peak. Exact errors with respect to the reported data are indicated in Table 1. The NSFD scheme seems to be substantially suited for the prediction of specific sizes of maxima during the course of the pandemic, as well as smooth increases in infection numbers. In contrast, the PINN based on the data and residual loss is able to capture smaller peaks and oscillations also within small time periods of a few weeks. The figure may lead to the view that the residual loss might not be crucial since high values of *α* result in better approximations, but Table 1 shows that a complete omission of the physics-informed part is not the best choice. The computation of the residual loss during the training procedure enables us to incorporate methodical information and knowledge about the disease, which solely data-driven methods do not include.

**Table 1:** Absolute and mean squared errors between the reported infection data and infection rate outputs of the PINN with training data covering the calendar weeks 10 in 2020 through 52 in 2021, or the NSFD scheme, if distinct weighting or transmission risk parameter values are used

size of *α* in PINN	diff (regularized by 10^−6^)	$\mathrm{MSE}_{\mathrm{diff}}$ (regularized by 10^−11^)
0.3	6.5338	0.64343
0.5	5.3861	0.57782
0.65	4.6681	0.53966
0.9	3.4886	0.48237
0.93	3.4015	0.47992
0.945	3.3726	0.47811
0.9475	3.3601	0.47772
0.95	3.3551	0.47601
0.9525	3.3599	0.47746
0.955	3.3717	0.47799
0.96	3.4683	0.48099
0.97	3.5714	0.48289
0.98	3.7614	0.49263
0.99	3.9904	0.49852

size of *β* in NSFD	diff (regularized by 10^−6^)	$\mathrm{MSE}_{\mathrm{diff}}$ (regularized by 10^−11^)
1.95⋅10^−9^	5.7471	0.71234
1.98⋅10^−9^	4.8266	0.50868
2.00⋅10^−9^	5.2971	0.62172
2.04⋅10^−9^	8.9822	1.58271
2.08⋅10^−9^	13.8549	2.63107
2.12⋅10^−9^	18.8948	6.80091

Figure 3 implies that a larger hyperparameter *α*, i.e., a greater influence of the data loss in the loss function leads to a better approximation of the peak heights. The height of the first local maximum in calendar week 47 is not captured, and it is predicted two weeks too early, but a larger *α* results in an improvement such that around 220,000 infections are predicted with $\alpha =0.95$. In this case, the NSFD scheme with $\beta =1.98e.9$ accounts for the actual peak height of around 400,000 (exactly: 403,329) weekly infections in the 91st regarded week. In contrast to the first, the second maximum of around 1.3 million weekly infections during the omicron phase is approximated very closely by our PINN if $\alpha >0.9$ is selected. This can be seen in the second and third visualized diagram. A strong increase is predicted at the end of the 94th regarded week, although it actually started in the 96th week, but the red an black curves approach each other until they meet in the 101st regarded week (calendar week 5, 2022). Weighting the two loss terms equally, i.e., setting $\alpha =0.5$, the second local maximum is predicted as only 800,000 infections such that there is a gap of 500,000 to the reported data. The behavior of the respective predicted curve in regard of increases and decreases is similar to the curve predicted using $\alpha >0.9$.

The local minimum after the second peak of the black curve is captured better by the NSFD scheme with $\beta =1.98\cdot 10^{-9}$ again. The PINN approach predicts a too small local minimum (625,000 with $\alpha =0.95$). However, the PINN is able to approximate the third peak (global maximum), whereas the NSFD scheme shows a very slight decrease again. This stresses that the PINN approach is more suited to account for quick changes in the data than the numerical discretization.

As a part of our validation process, we computed the absolute difference between the reported data and compartment size output of the PINN as

27 $$ \mathrm{diff} = \sum_{j=1}^{l} \mathrm{diff} _{j} , $$

where

28 $$ \mathrm{diff} _{j} = \bigl\Vert \hat{\mathcal{K}}^{j}-{ \mathcal{PINN}_{p}^{W}}_{j} \bigr\Vert \quad \forall j=1,\dots , l , $$

and the mean squared error as

29 $$ \mathrm{MSE}_{\mathrm{diff}} = \frac{1}{l}\sum _{j=1}^{l} {\mathrm{diff} _{j}}^{2} , $$

where *l* is the number of weeks for which the output of the PINN, and the reported data shall be compared.

In Table 1, the errors for the validation runs of the infection rate prediction are expressed for distinct assignments of the weighting parameter *α*. We selected $l=29$ to compare the reported and predicted infection numbers between the 34th calendar week in 2021 and the 10th calendar week in 2022. The parameter *α* does not occur in the NSFD estimation since it weighs the two loss functions terms of the PINN, and in the PINN approach, the parameter *β* is estimated so that it does not occur as a parameter of fixed size here.

Table 1 proves that the size of both errors increases the more the smaller *α* is selected compared to 0.9. We obtain values of diff less than $3.5\cdot 10^{-6}$ if we choose $\alpha \in [0.9,0.96]$ and values of $\mathrm{MSE}_{\mathrm{diff}}$ less than $0.5 \cdot 10^{-11}$ if we choose $\alpha \ge 0.9$. We can observe that both errors increase again for $\alpha >0.95$ here. The errors for $\alpha =0.93$ are somewhat smaller than for $\alpha =0.96$, and the errors for $\alpha =0.9$ are smaller than for $\alpha =0.99$. This indicates that a small decrease in the computed errors can be achieved by weighting the loss $\mathrm{MSE}_{\mathcal{F}}$ 5% instead of 0% (or, e.g., 7% instead of 3%) in the loss function. A weight of 0 for $\mathrm{MSE}_{\mathcal{F}}$ would be given if we did not include any residual loss.

We are regarding a specific scenario with 43 weeks as the training basis and 29 weeks as the validation basis. As already seen in Fig. 3, assigning $\beta =1.98\cdot 10^{-9}$ in the NSFD scheme yields a predicted curve that meets the local maximum in the 91st regarded week and the local minimum in the 104th/105th regarded week. The obtained error diff is almost half as small as for $\beta =2.04\cdot 10^{-9}$, and $\mathrm{MSE}_{\mathrm{diff}}$ is more than 3 times smaller. The total difference diff is slightly larger than for the PINN with $\alpha =0.65$ and smaller than for the PINN with $\alpha =0.5$.

Regarding the errors diff for $\beta =2.08\cdot 10^{-9}$ or $\beta =1.12\cdot 10^{-9}$ (13.8549 or 18.8948, respectively), it is obvious that a good choice of the transmission risk parameter is highly relevant for the performance of the NSFD in the forecast. We modified the transmission risk manually here to show the effect on the approximation quality. There exist optimization algorithms in which model parameters are optimized during a process of minimization of the error between reported and numerically predicted data. Our focus is the comparison of PINN predictions with NSFD forecasts based on our compartment model with selected transmission risks in this paper. The NSFD method is eligible for approximating a segment of reported COVID-19 data, substantially a local or global peak. The error reduction is extremely dependent on the parameter choice. As described in the analysis of Fig. 3, the PINN approach seems to be much better suited than the NSFD scheme to predict local fluctuations observed in the reported data and make short-term predictions.

**Figure 4:**
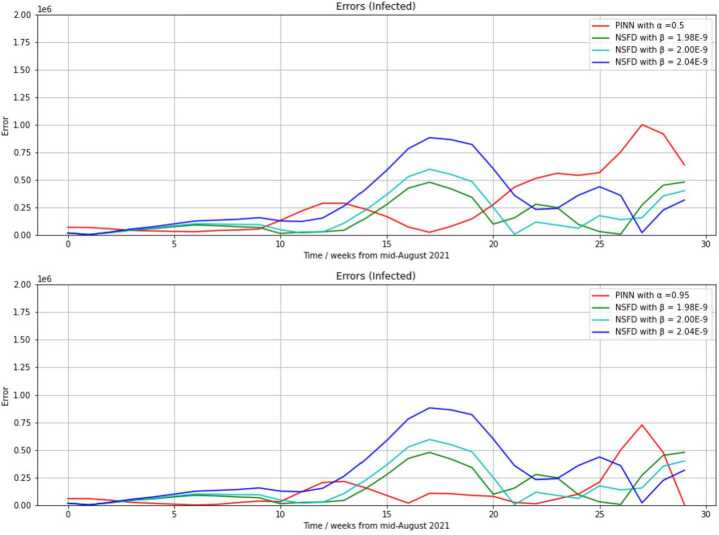
Errors between the reported data and the infection numbers obtained with the NSFD scheme with three different assignments of the transmission risk parameter *β*, and the PINN with weighting parameter $\alpha =0.5$ (upper diagram) or $\alpha =0.95$ (lower diagram), and for the 29 calendar weeks starting from August 23rd 2021 (34th calendar week in 2021)

Figure 4 shows the total differences $\mathrm{diff} _{j}$ (following: *errors*) between the reported COVID-19 infection rates and predictions with the PINN and NSFD corresponding to Fig. 3.

The visualization of the differences to the reported data resulting from the choices $\alpha =0.5, 0.95$ or $\beta =1.98\cdot 10^{-9}$, $2.00 \cdot 10^{-9}$, $2.04\cdot 10^{-9}$ in Fig. 4 verifies the corresponding results of Table 1. The errors of both methods with any choice of *α* or *β* is comparatively small, i.e., below $0.2\cdot 10^{6}$, during the first 10 predicted weeks. We must take into account that the prediction refers to the time at which the highest infection rates experienced during the whole COVID-19 pandemic occurred. Consequently, prediction errors will be larger than in other time periods since the outbreak of COVID-19 that could be regarded.

It can be seen that the niveau of the error of the NSFD with $\beta =1.98\cdot 10^{-9}$ remains below $0.5\cdot 10^{6}$ throughout the 29 weeks, whereas an error peak of $0.88\cdot 10^{6}$ is reached in the 17th regarded week if we use $\beta =2.04\cdot 10^{-9}$. With a higher transmission risk, the NSFD achieves a higher predicted peak.

Data until the third and global maximum of $1{,}571{,}595$ weekly infections are considered, and $\beta =2.04\cdot 10^{-9}$ is the only transmission risk choice among the three visualized choices, which yields a peak of 1.5 million weekly infections. Thus, the blue curve ($\beta =2.04 \cdot 10^{-9}$) in Fig. 4 is on the lowest level from the 27th regarded week. We obtain comparatively very small errors with the NSFD scheme at times at which peaks are attained in the reported data.

The error between the NSFD predictions and the reported data is largest at the local minimum between the first and second omicron wave around calendar week 52 in 2021. The error between the PINN prediction and the reported data is largest at the local minimum between second and third omicron wave around calendar week 9 in 2022.

The relatively large error of ⋅10^6^ with $\alpha =0.5$ or $0.73 \cdot 10^{6}$ with $\alpha =0.95$ (PINN approach) in the 28th regarded week is reasoned by the great difference to the reported data (black curve) in the calendar weeks 9 and 10 in 2022 in Fig. 3. The local minimum is predicted as too small by the PINN here. Nevertheless, the PINN actually predicts a local minimum, whereas the NSFD approach predicts a maximum or slightly decreasing infection rates right after a reached maximum here.

Altogether, the PINN with $\alpha =0.95$ yields the smallest error level among the five parameter selections ($\alpha =0.5,0.9$, $\beta =1.98\cdot 10^{-9}$, $2.00\cdot 10^{-9}$, $2.04\cdot 10^{-9}$) during the 29 regarded weeks. It remains below $0.25\cdot 10^{-6}$ until the 25th regarded week.

### Validation process: hospitalization rates

The results of the prediction of hospitalization rates are discussed in the sequel. Hospitalization numbers resulting from the application of the NSFD scheme are not included here because we focus on the PINN, which is the main method of this paper. The NSFD scheme applied to another compartment model used for the COVID-19 pandemic data is described in detail in [27]. Table 2 displays the errors defined in Equations (27) and (29) for the validation runs of the hospitalization rate prediction for distinct assignments of the weighting parameter *α*. We selected $l=35$ to compare the reported and predicted hospitalization numbers between the 31st calendar week in 2021 and the 14th calendar week in 2022.

**Table 2:** Absolute and mean squared errors between the reported hospitalization data and hospitalization rate outputs of the PINN with training data covering the calendar weeks 10 in 2020 through 52 in 2021 if distinct weighting parameter values are used

size of *α*	diff (regularized by 10^−5^)	$\mathrm{MSE}_{\mathrm{diff}}$ (regularized by 10^−8^)
0.3	4.7669	1.990189
0.5	3.6339	1.291875
0.65	3.6018	1.273610
0.9	2.0726	0.783272
0.93	1.7738	0.704100
0.94	1.3623	0.310871
0.945	1.1439	0.180857
0.9475	1.2032	0.230114
0.95	1.4719	0.415736
0.9525	1.5091	0.448027
0.955	1.5917	0.602143
0.96	1.7551	0.688926
0.97	2.2789	0.858422
0.98	2.7267	0.925258
0.99	2.7442	0.935908

As can be seen in Table 2, the PINN performance significantly improves for values $\alpha >0.9$, but deteriorates for $\alpha >0.96$. This is a similar result as in Table 1, where the best manually generated result was obtained with the assignment $\alpha =0.95$.

In the forecast of hospitalization numbers, we obtain smaller errors than in Table 1 since the number of weekly hospitalizations is much smaller than the number of weekly infections. The peak in hospitalizations during the previous course of the COVID-19 pandemic was 12,658 weekly hospitalizations in the calendar week 51 in 2020. The highest peak since August 2021, i.e., during the time of dominance of the omicron variant, was 11,153 in the calendar week 11 in 2022.

It can be noted that the smallest error diff in Table 2 is $1.1439\cdot 10^{-5}$, and the smallest error $\mathrm{MSE}_{\mathrm{diff}}$ is $0.180857 \cdot 10^{-8}$. Both are achieved for $\alpha =0.945$. The error diff for $\alpha =0.945$ is only 31% of the error obtained with $\alpha =0.5$ and 24% of the error generated with $\alpha =0.3$.

The error diff for $\alpha =0.93$ is 20.7% larger than for $\alpha =0.95$, whereas the error diff for $\alpha =0.9$ is 24.5% smaller than for $\alpha =0.99$. In the prediction of hospitalization rates through our PINN, we can thus notice a clearer disadvantage in neglecting the residual loss, i.e., setting $\alpha =1$, than in the forecast of infection rates.

**Figure 5:**
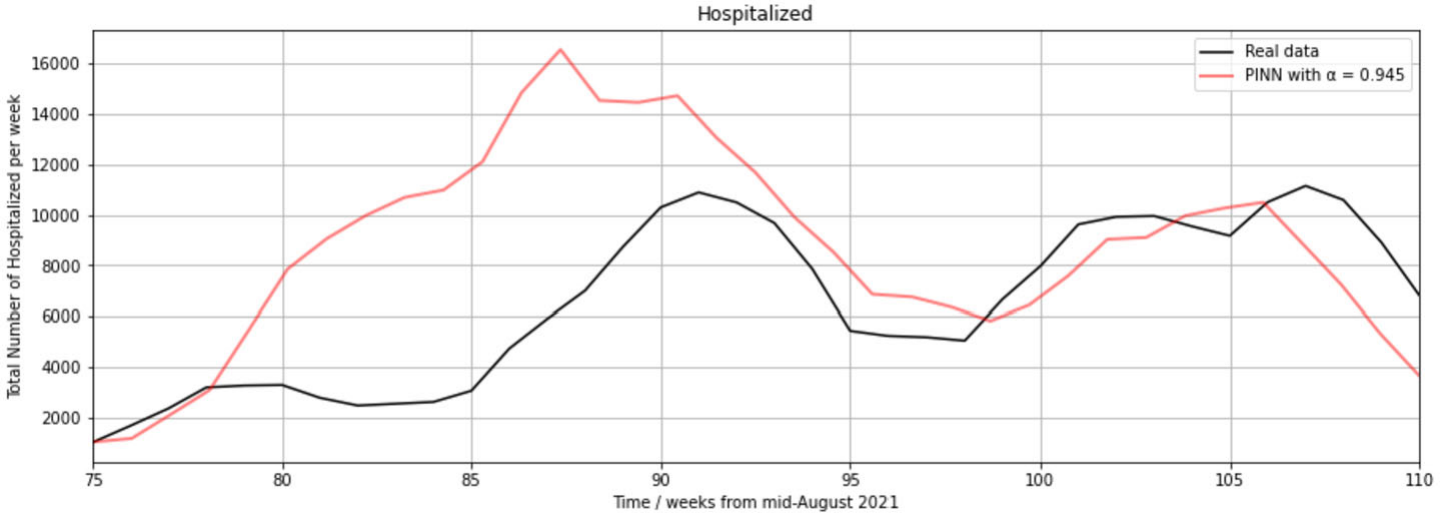
Hospitalization numbers obtained from the reported data (black) or the training of the PINN (red) from the 75th regarded week (31st calendar week in 2021) for $\alpha =0.945$

Figure 5 shows the hospitalization rates between August 2021 and April 2022 predicted by the PINN with $\alpha =0.945$ compared to the reported data corresponding to this time period.

The increase in hospitalization between the 75th and 91st regarded week (calendar weeks 31 through 47, i.e., from 2 August to 28 November 2021) has a peak at 10,894 weekly hospitalizations. The PINN also predicts an increase at that time, but a stronger increase peaking at 16,500 hospitalizations in the 87th regarded week. A small decrease of 673 weekly hospitalizations is visible in the reported data between the 80th and 84th regarded week, after which the curve rises stronger than before (1306 weekly hospitalizations between calendar weeks 41 and 47). The prediction of the PINN shows a flattening in the 83rd and 84th regarded week. It also displays an increase of 1250 weekly hospitalizations between the 81st and 87th regarded week. In the 91st regarded week, the PINN curve exhibits a local maximum of almost 15,000, which is almost 4000 too many hospitalizations.

Between the 91st and 98th regarded week (calendar weeks 47 in 2021 through 4 in 2022), the PINN curve approaches the curve of reported data and crosses it at 6662 infections in the calendar week 3 in 2022. Further overlapping points are 9957 hospitalizations in the 103rd and 9924 hospitalizations in the 106th regarded week, which occurs during an increase in hospitalization numbers in both the reported and predicted data between the 98th and 107th regarded week, with a short decrease of 374 hospitalizations visible in the reported data between the 104th and 105th week.

In the prediction of the PINN, the 9924 hospitalizations in the 106th regarded week define a maximum, whereas a local peak of 11,153 hospitalizations is observable in the reported data one week later. Both curves decline with approximately the same slope after reaching their respective peak. Subsequently, the curve generated with the reported data ends at 6813 hospitalizations in the 110th regarded week (calendar week 14 in 2022) and the predicted curve terminates at 4000 hospitalizations.

With respect to that, the error effected by the PINN in this scenario attains a maximal value of 9500 in the 86th regarded week. It becomes minimal in the 77th, 10th, and 106th regarded week, when the two curves cross each other.

### Infection scenario predicted by the PINN

**Figure 6:**
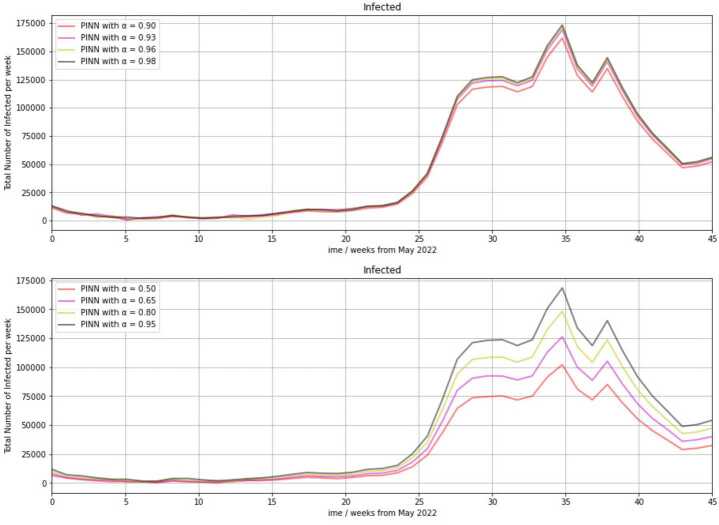
Forecast of infection numbers (size of the infected compartment) from the end of May 2022 with the PINN

We finally let the PINN predict infection numbers with training data covering the calendar weeks 10 in 2020 through 14 in 2022. We used $\alpha =0.95$ for the weighting parameter. The calendar week 14 in 2022 ended on 10 April 2022. The RKI registered 970,745 weekly infections in that week. Due to the clear downward trend since 31 March 2022 visible in the latest data, we assumed a strong decline in infection numbers until the end of May 2022 [16]. In the calendar week 22 in the year 2021, 20,689 new infections were registered by the RKI [18]. Our following prediction starts at the end of May 2022. Figure 6 visualizes scenarios generated with our PINN with seven distinct selections of the weighting parameter.

We can see in both diagrams in Fig. 6 low infection rates are predicted for the whole summer of the year 2022. The weekly rates are below 20,000 during the calendar weeks 22 through 42 in 2022. Sharply increasing infection numbers are forecasted from November 2022, and a maximum is predicted in late January 2023. This is the same for all of the shown selections of the parameter *α*. We can see in the lower diagram, that almost 175,000 weekly infections are obtained at the peak with $\alpha =0.95$, which is the value of *α* that yielded the best approximation in the validation part. The number of infections is similar to the calendar week 44 in 2021, when 177,889 infections were registered by the RKI.

The lower diagram implies a great range in infection rates between choices of *α* between 0.5 and 0.95, whereas the upper diagram indicates a very small range between choices of *α* between 0.9 and 0.98 in the PINN approach. This corresponds to our results from Table 1. Using $\alpha =0.5$, i.e., a 50% inclusion of the residual loss in the loss function, the predicted maximum is only 100,000 infections. With $\alpha =0.65$, the peak is 125,000, and with $\alpha =0.80$, it is 148,000 infections. Over the course, a range between the curves of up to 50,000 is observable. In contrast, the maximal range between any two curves in the upper diagram is 10,000. It occurs in the calendar week 45.

After a local peak forecasted as 138,000 infections with $\alpha =0.95$ in February 2023, infection rates are predicted to decline almost as sharply as they increased, reaching a local minimum at the beginning of April in 2023. This minimum is predicted to be 5000 infections if $\alpha =0.95$ is used as the value of the weighting parameter. Despite the inclusion of the two omicron-induced maxima of February and March 2022 (1,307,475 and 1,571,595 weekly new infections), our PINN does not predict as high infection numbers for the end of the year 2022. To be able to better classify the prediction, it has to be considered that the maximal infection rate during the second half of the year 2021 was 403,329 infections in the last week of November, and the maximum during the first half of the year 2021 was 145,494 in the first week of January. It also be minded that a constant vaccination rate is assumed in the model.

Aside from prediction with the PINN and the NSFD scheme in [27], we made a forecast with the NSFD scheme. As an addition to our previous NSFD implementations, we used a time-dependent function $\beta (t)$ for the transmission rate. This function has got the trigonometric shape

30 $$ \beta (t) = \beta _{0} \biggl(1+c \sin \biggl( \frac{t+t_{0}}{\frac{\pi}{2}} \biggr) \biggr) . $$

Here, $\beta _{0}$ is the initial transmission risk. It was set $\beta _{0} = 1.99\cdot 10^{-9}$, and we assigned $t_{0}=25$. The parameter *c* is responsible for the amplitude of the resulting function. It was modified, as the fixed parameter *β* in our previous implementations to obtain different predictions. Using $c=0.03, 0.05$ or 0.07, we obtain a maximal value of 2.0497, 2.0895, or 2.1293 of $\beta (t)$, respectively, and a minimal value of 1.9304, 1.8907, or 1.8509. The first two minima of $\beta (t)$ are reached 5 and 45 weeks and the first maximum is attained 25 weeks after the observation start, i.e., time $t_{0}$.

**Figure 7:**
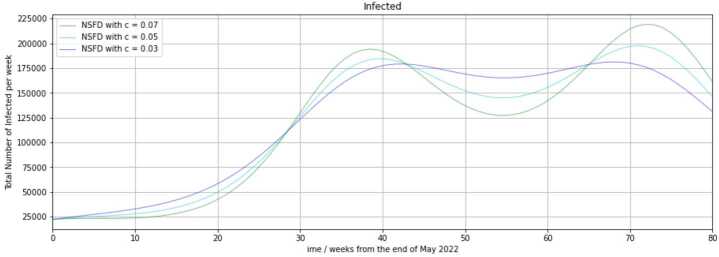
Forecast of infection numbers (size of the infected compartment) from the end of May 2022 with the NSFD scheme

Figure 7 shows a prediction of infection rates of the NSFD scheme from the end of May 2022.

As shown in Fig. 7, the time-variability with a sine function in the transmission rate leads to more than one peak over the course of time. Nonetheless, only one peak is detectable per wave, as in our previous predictions with the NSFD scheme. Using $c=0.07$, we have maxima in the 38th and 72nd regarded week, which correspond to late February and late October 2023. With $c=0.05$, the maxima are reached in the 39th and 71st regarded week. Using $c=0.03$, the peaks are attained in the 42nd and 70th regarded week. It is interesting to note that the second reached peak is higher than the first one in all three cases, but the difference in peak height is approximately 30,000 for $c=0.07$, 15,000 for $c=0.05$, and 3000 for $c=0.03$. Moreover, the first observed peak is achieved at a similar time as in the prediction of the PINN in Fig. 6, where it was reached in the 34th regarded week. If we use $\alpha =0.95$ in the forecast of the PINN, the peak in Fig. 6 with a size of almost 175,000 is on a similar level as the first observable maximum in the given predictions with the NSFD scheme in Fig. 7. Their heights are approximately 190,000 with $c=0.07$, 185,000 with $c=0.05$, and 182,000 with $c=0.03$. Figure 7 illustrates that a local minimum is attained in the 54th regarded week, i.e., in early June 2023. This is the case for all three assignments of the parameter *c*. If we set $c=0.03$, the is reached at approximately 168,000 weekly infections. Using $c=0.05$, the reached value is 145,000. With $c=0.03$, we obtain only 127,000 weekly infections at the minimum.

## Conclusion and outlook

We have presented a data- and physics-driven deep learning algorithm that identifies the transmission parameter *β* and the parameter *κ*, which represents the proportion of transmissibility in the vaccinated population, in a system of ordinary differential equations of a compartmental model describing population dynamics at pandemic times. For this purpose, we used a physics-informed neural network (PINN) whose loss function combines a weighted data loss with a weighted residual loss, which in turn is based on the compartment model applied to the respective current network output.

Using COVID-19 infection, hospitalization, vaccination, and mortality data from Germany, we calculated the prediction error of a non-standard finite difference scheme (NSFD) [13] and the PINN we developed. During the validation process, we slightly adjusted the parameters of the NSFD scheme and the network architecture of the PINN so that we obtained resulting plots that approximate the trend of the real course of infection numbers.

Depending on the choice of the transmission rate, the performance of the NSFD scheme varies in approximating the course of the pandemic. Slight changes can lead to large changes in the prediction in terms of the slope and the height reached by the peak of the curve. For example, a $4 \cdot 10^{-10}$ increase in transmission risk from $2.00\cdot 10^{-9}$ to $2.04\cdot 10^{-9}$ resulted in a maximum reached two weeks earlier and $0.32\cdot 10^{6}$ higher, and a $2\cdot 10^{-10}$ decrease in risk from $2.00\cdot 10^{-9}$ to $1.98\cdot 10^{-9}$ resulted in a maximum reached one week later and $0.9\cdot 10^{5}$ smaller (see Fig. 3). The scenarios drawn in our validation part can be described as *long-term predictions* in the way that data from the outbreak of the pandemic in Germany until the end of 2021 are used in the training set. We did not include data from just a single peak within a wave or a single wave here, as we wanted an algorithm that was adapted to a wide range of virus variants. It should also be adapted to the behavior of SARS-CoV-2 in all four seasons, which would not be given if only one wave or even one local peak were included in the training data.

PINN seem better suited to account for multiple local maxima within a wave than the NSFD scheme, as it is able to capture outliers or small oscillations in a given set of real infection or hospitalisation data and transfer them into its prediction. The clear advantage of PINN over discrete numerical methods is its data orientation, so that it does not rely solely on a mathematical model described by a system of differential equations, which certainly cannot capture all aspects or dynamics of infectious disease. The importance of data loss is evidenced by the fact that predictions of our PINN with assignments $\alpha >0.9$, i.e., a clearly dominant influence of the data error $\mathrm{MSE}_{\mathcal{U}}$, led to resulting curve predictions that were closer to the reported data of the 30 weeks considered than with assignments $\alpha <0.9$. Indeed, our results, particularly those in Table 1, show a sharp decline in PINN performance for decreasing values of the weighting parameter.

With regard to the prediction of the NSFD scheme, the inclusion of time variability in the transmission rate showed that the NSFD scheme is able to accommodate the prediction of more than one wave, each with a maximum. It is important that the parameters used in the time-dependent transmission function are chosen appropriately. If the NSFD scheme were to be used for an actual prediction of the next wave of the COVID-19 pandemic, the parameters *c* would need to be reliably estimated.

Nevertheless, the residual term allows us to incorporate systematic knowledge about the spread and transmission dynamics of the disease into our neural network. The differential equations make the network a physically informed and partially mathematical approach. No mathematical model is able to perfectly describe an infectious disease such as COVID-19 because, for example, new mutant variants emerge and intervention measures or population compliance change over time, and not every epidemiological detail relevant to transmission is known. However, the values of certain fixed parameters can be derived from elaborate studies, knowledge of a possible next mutation, intervention, or vaccination strategy can be incorporated, and SIR-type models provide clues to transition dynamics that the neural network can work with. Our variant of the SIR model, i.e., the SVIHR model, was developed because we wanted to include leaky vaccination to account for the reduced infectiousness of individuals in the fully vaccinated case, and we wanted to focus on predicting the number of hospitalizations as an important part of pandemic-related policy and hospital occupancy planning.

We found similar best values for the residual and data loss weight parameter when observing infection and hospitalization rates. Our results show the best performance of PINN for $\alpha =0.95$ in terms of predicting infection rates and $\alpha =0.945$ for predicting hospitalization rates, implying a non-negligible weight of 5% and 5.5%, respectively, of residual loss in the loss function used during the training procedure.

We briefly remark that an even more accurate way to compute the denominator function would take into account the transition rate $\Upsilon _{i}$ at which the *i*th compartment is entered by individuals for all model compartments $\mathcal{K}_{i}$, $i=1,2,\dots $ [6]. In this case, the parameter *μ* occurring in the denominator function in Equation (26) would be replaced by a parameter $1/T^{*}$. $T^{*}$ could be determined as the minimum of the inverse transition parameters:

$$ T^{*} = \min_{i=1,2,\dots} \biggl\{ \frac{1}{\Upsilon _{i}} \biggr\} . $$

In our future work, time-varying functions for vaccination and transmission rate will be included to account for seasonal and variant-dependent fluctuations, cf. [8]. In addition, the prediction of hospitalization rates can be refined by including more precise knowledge than the transmission risk, the hospitalisation rate, and the average length of stay in a hospital. For example, the Bayesian method could be a tool to estimate the weekly demand for hospital beds. We will also incorporate an adaptive learning rate into our code.

## Data Availability

The authors used freely available data from RKI.
